# Exploring the Relationships of Financial Literacy and Financial Behaviour with Health-Related Quality of Life (HRQOL) among the Low-Income Working Population in Malaysia during the COVID-19 Pandemic

**DOI:** 10.3390/ijerph191912520

**Published:** 2022-09-30

**Authors:** Mohamad Fazli Sabri, Mas Ayu Said, Amirah Shazana Magli, Tan Maw Pin, Hussein Rizal, Nithiah Thangiah, Muslimah Ithnin, Hazreen Abdul Majid, Rozmi Ismail, Tin Tin Su, Abdul Rahim Husniyah

**Affiliations:** 1 Department of Resource Management and Consumer Studies, Faculty of Human Ecology, Universiti Putra Malaysia, Serdang 43400, Malaysia; 2 Department of Social and Preventive Medicine, Faculty of Medicine, University of Malaya, Kuala Lumpur 50603, Malaysia; 3 Centre for Epidemiology and Evidence-Based Practice, Department of Social and Preventive Medicine, Faculty of Medicine, University of Malaya, Kuala Lumpur 50603, Malaysia; 4 Department of Medicine, Faculty of Medicine, University of Malaya, Kuala Lumpur 50603, Malaysia; 5 Centre for Population Health c/o, Department of Social and Preventive Medicine, Faculty of Medicine, University of Malaya, Kuala Lumpur 50603, Malaysia; 6 Corporate Communication Unit, Ministry of Health Malaysia, Putrajaya 62000, Malaysia; 7 Department of Nutrition, Faculty of Public Health, Universitas Airlangga, Surabaya 60115, Indonesia; 8 Psychology and Human Wellbeing Research Centre, Faculty of Social Sciences and Humanities, Universiti Kebangsaan Malaysia, Bangi 43600, Malaysia; 9 South East Asia Community Observatory (SEACO) & Global Public Health, Jeffrey Cheah School of Medicine and Health Sciences, Monash University Malaysia, Subang Jaya 47500, Malaysia

**Keywords:** financial literacy, financial behaviour, health-related quality of life, low-income, pandemic

## Abstract

This cross-sectional study examined the relationships of financial literacy (FL) and financial behaviour (FB) with health-related quality of life (HRQOL) during the COVID-19 pandemic among low-income working population (20–60 years old) in Malaysia. A self-administered questionnaire survey was used with HRQOL data were gathered using the EuroQol 5-Dimension 5-Level (EQ-5D-5L) tool. A generalised linear model was employed to examine the hypothesised relationships between the constructs. From 1186 respondents, the majority were employed (73.9%), had a monthly household income of less than RM 2500 (74.5%), and did not have any chronic medical conditions (74.5%). The mean (SD) values of FL, FB, and EQ-5D-5L were 5.95 (1.48), 22.08 (4.79), and 0.96 (0.10), respectively. The results of the adjusted model revealed lower age group, Malay ethnicity, Indian ethnicity, and increased FB score as significant determinants of higher EQ-5D-5L scores. With the addition of the chronic medical condition factor into the saturated model, the lower age group, ethnicity, and no chronic medical condition were significant determinants of higher HRQOL. The effects of FB on QOL were confounded by chronic diseases, implying that interventions that focus on improving FB for those with chronic medical condition may help to improve the QOL among the low-income working population.

## 1. Introduction

The spread of severe acute respiratory syndrome Coronavirus 2 (SARS-CoV-2) has led to the COVID-19 pandemic worldwide, which was first declared by the World Health Organisation (WHO) on 11 March 2020 [1]. The virus rapidly spreads through respiratory droplets, as well as direct or indirect contact with the eyes, mouth, and nose [2]. Following the recommendations of the WHO, the Malaysian government enforced the Movement Control Order (MCO) nationwide to curb the spread of the virus on 18 March 2020. Only 22 economic sectors were allowed to operate during the first phase of MCO from 18 March 2020 to 31 March 2020, and the number of operating sectors was reduced to only 10 during the second phase of the MCO, which was extended to 14 April 2020. The Malaysian government introduced various measures to curb the transmission of the virus, such as physical distancing, movement restrictions, international travel ban, school closure, and work-from-home practice [3].

However, the nationwide lockdown led to a significant decline in household income. According to the Department of Statistics Malaysia (DOSM), the recorded number of low-income households increased from 405,400 in 2019 to 639,800 in 2020. Furthermore, both incidence of absolute poverty (5.6% in 2019) and incidence of hardcore poverty (0.4% in 2019) increased to 8.4% and 1.0%, respectively, in 2020 [4]. The enforcement of MCO resulted in recurrent economic activities, which affected many Malaysians, especially the low-income population [5,6]. Most businesses had their employees work from home, and financially affected employers had to terminate the employment of their employees [7]. With the new norms, all Malaysian households have to make additional purchases of face masks, disinfectants, and COVID-19 self-test kits [8]. All these individual and community-based measures taken by the Malaysian government have successfully reduced the number of COVID-19 cases; but this has substantially affected Malaysian households, especially the vulnerable low-income population, in terms of their health and socio-economic status [9].

With respect to the Sustainable Development Goals (SDGs) of the 2030 Agenda for Sustainable Development (2030 Agenda), particularly on the principle of “leaving no one behind”, the Malaysian government has made efforts to drive the local implementation of SDGs for the country’s development towards the realisation of the Shared Prosperity Vision 2030 (WKB 2030): to provide a decent standard of living by 2030 for all Malaysians [10]. With that, the government introduced the Twelfth Malaysia Plan (12MP) 2021–2025, acknowledging the goal of improving all living standards, especially for the low-income population [11]. WHO also acknowledges the importance of evaluating and improving the quality of life (QOL) in all facets of human life [12,13]. Focusing on health and illness, QOL or commonly known as health-related quality of life (HRQOL) serves as an important metric in healthcare and public health interventions.

There are many instruments available to measure HRQOL, including the EuroQol 5-Dimension 5-Level (EQ-5D-5L) tool, which was specifically used in this study [14,15]. The EQ-5D was developed to describe and value health across a wide range of disease areas. They are also frequently used in research into health in the general population. Aside from its robust validity and reliability, the tool is short and uses simple language, making it easy to answer and suitable for widescale use among Malaysians [16,17,18]. The incorporation of physical, mental, emotional, and social functions in this widely used tool measures the overall well-being and health-related satisfaction [16,17].

Prior studies noted the significant impact of the MCO enforcement on Malaysians, especially among the low-income population [6,19,20]. It is crucial for the government to comprehend how financial literacy and financial behaviour affect the HRQOL of low-income population given their significant struggle during the pandemic. According to the active ageing framework, enhancing QOL as people age requires optimising opportunities of health, participation, and security [21]. Early investments in health, social participation and financial stability may lead to better QOL. By having financial security, households would be better prepared to face unforeseen circumstances, for instance, medical emergencies or unemployment; as well as provide a buffer to protect themselves from loss of income.

Realising the need to mitigate the adverse effects of the COVID-19 pandemic, the Malaysian government introduced a financial stimulus package as financial aid for the vulnerable low-income population, which was highlighted to be inadequate [19]. Thangiah et al. found poor QOL and HRQOL among low-income households in Malaysia [20]. Meanwhile, a few prior studies linked low-income populations to a low level of financial literacy and identified financial behaviour as a significant predictor of financial well-being [22,23]. Studies have also highlighted the relationship of lower level of financial literacy with lower earnings and savings and how these relationships adversely affect the population’s health, well-being, and QOL [19,23].

Garey et al. [24] investigated the relationship between financial strain and HRQOL among respondents in the United States. They discovered that tremendous financial stress and less social support were associated with lower HRQOL. On the other hand, Tamson et al. [25] examine HRQOL among the general population of Estonia and its socio-demographic and behavioural correlates during the COVID-19 pandemic. In baseline and follow-up measures, older age, unemployment or economic inactivity, and financial difficulties were substantially linked with lower HRQOL. In contrast, Gildner et al. [26] discovered that older adults in China and Russia were more likely to report adequate income than their 18 to 49-year-old counterparts. In contrast, the opposite trend was observed in Ghana and India. Findings indicated a connection existed between self-related health (SRH), quality of life (QOL), and financial sufficiency.

Consequently, research on financial behaviour and HRQOL is relatively scant. Thus, it is imperative to have a deeper understanding of the relationship between financial literacy and financial behaviour with HRQOL among low-income households to provide a comprehensive understanding of how to improve HRQOL among the low-income working population. As a result, scholars could examine QOL from various angles and perspectives; therefore, various nations could characterise and evaluate the QOL using distinct variables.

In view of the above, this study aimed to examine the relationships of financial literacy and financial behaviour with HRQOL among the low-income working population in Malaysia. This study hypothesised that low-income working population has a low level of financial literacy and poor financial behaviour, resulting in poor HRQOL during the COVID-19 pandemic. The negative implications of financial constraints on HRQOL potentially worsen the long-term QOL among the low-income population, which clearly justified the need of the current study to explore the influence of financial literacy and financial behaviour on HRQOL for this population. Although the adverse effects of the COVID-19 pandemic on the financial and health aspects may be irreversible for this vulnerable population, such findings can help the Malaysian government to formulate a better strategy for the country’s future health policy agenda as the nation progresses to its endemic phase.

## 2. Materials and Methods

### 2.1. Study Design and Duration

Accordingly, there are three main categories of household income in Malaysia: (1) the bottom 40% (B40) (household monthly income of lower than RM 4850); (2) the middle 40% (M40) (household monthly income of between RM 4851 and RM 10,960); (3) the top 20% (T20) (household monthly income of more than RM 10,960). This cross-sectional study focused on low-income working Malaysians (B40 group) of between 20 and 60 years old. The data collection process was conducted from 1 September 2020 to 24 October 2020 (first interval) and 25 January 2021 to 15 February 2021 (second interval) due to the enforcement of MCO that restricted all movements.

### 2.2. Instrument

Three public health experts and two personal finance experts were appointed to examine the content validity of the developed instrument. The instrument consisted of six sections, which focused on socio-demographic, socio-economic, health-related, financial literacy, financial behaviour, and HRQOL questions. In particular, respondents were first required to indicate their age, gender, ethnicity, marital status, education level, employment status, and monthly household income. They were also required to state if they have any chronic medical condition(s) and list their condition(s) if there is any.

Next, the instrument included ten financial literacy questions with “yes” and “no” responses, which covered general financial knowledge, credit cards, debts and loans, savings, and investments (Appendix A). These questions were adapted from previous literature to fit the Malaysian context, specifically the B40 group [27,28]. Every correct response was coded as one point, whereas each incorrect answer was coded as zero points. A total financial literacy score was then calculated to gain a total financial literacy score. Poorer scores imply lower financial literacy, whereas higher scores indicate greater financial literacy. This method was also implemented by Compen et al. [29] and Johan et al. [30], which suggest that it would provide a greater understanding of how financial literacy affects HRQOL.

The instrument also included ten questions on financial behaviour, which were adopted from Kapoor et al. and Hogarth and Hilgert [31,32] (Appendix B). Respondents were required to reveal their views on financial planning in terms of financial goals, cash management, credit management, investments, and savings according to a four-point Likert scale, with the endpoints of “never” and “always”. Next, a total financial behaviour score was calculated by summing up the ten items; a lower score reflects poorer financial behaviour (Cronbach’s alpha = 0.879). The same technique was implemented by Nano [33] and Hu and Feng [34] to investigate the differences in financial behaviour and their impact [1].

As previously noted, the EQ-5D-5L tool was employed in this study [14]. Using this tool, HRQOL was measured in terms of mobility, self-care, usual activities, pain/discomfort, and anxiety/depression (Appendix C). For every listed dimension, respondents were required to describe their current health status according to the following five-point Likert scale (severity scale): (1) Having no problems; (2) Having slight problems; (3) Having moderate problems; (4) Having severe problems; (5) Unable to/having extreme problems. The Malay version of the EQ-5D-5L tool was validated in the general population and target group settings [18].

### 2.3. Data Collection

Referring to the DOSM database, this study randomly identified Malaysians of between 20 and 60 years old with low household monthly income (of less than RM 4850) [27]. Each of the four regions in Peninsular Malaysia was represented by a specific state—Selangor state represented the country’s central region; Pahang state represented the eastern region; Johor state represented the southern region; the state of Pulau Pinang represented the northern region. Meanwhile, for East Malaysia, both states of Sabah and Sarawak were included in this study. This study randomly sampled the participating municipalities in each respective state. Data was collected through face-to-face self-administered questions by a survey interviewer. In this case, 71 trained enumerators administered and facilitated the survey under a research coordinator’s supervisor. As a result, a total of 1186 respondents were successfully recruited for the questionnaire survey.

As of 2020, Malaysia has a population of 32.66 million with a total of 8.2 million households. The number of households with a low-income category B40 was determined to have climbed from 405,00 in 2019 to 639,800 in 2020 [4]. Furthermore, according to Krejcie and Morgan’s [35] sampling size calculation table, the required sample size for a population of one million or more is approximately 384 samples, with a 95% confidence interval and a 2.5% margin of error. This justification was supported by Hair et al. [36], Kline [37], Sekaran [38], and Dillman [39], who argued that sample size must be proportional to the study context and representative of the population. Furthermore, the sample size is calculated based on various factors and criteria, such as model complexity, the estimated rate of missing data, and the analytical procedures employed [36]. Henceforth, a deliberate sample size of 1186 is acceptable and sufficient for this analysis, providing that the sample represents the B40 low-income population income group.

### 2.4. Data Analysis

Frequency analysis was performed to identify any missing values and extreme values. All cases of extreme values resulting from incorrect data entry were removed and substituted with missing values. Following that, the obtained data were first examined descriptively before the study proceeded to bivariate analysis.

This study included ten domains for independent variables: age, gender, ethnicity, marital status, education level, employment status, monthly household income, chronic medical condition, financial literacy, and financial behaviour. Data for age, financial literacy, and financial behaviour were continuous data. Meanwhile, data for gender, employment status, and monthly household income were grouped into two groups of categorical data, whereas data for marital status and the presence of chronic medical condition were grouped into three groups of categorical data. Data for ethnicity and education level were grouped into five groups of categorical data. Therefore, the values of mean and standard deviation were obtained for continuous data, while numbers and percentages were obtained for categorical data.

Meanwhile, HRQOL outcomes were measured based on the EQ-5D-5L scores. Firstly, based on the results of the Shapiro-Wilk test for normality, EQ-5D-5L scores were not normally distributed (*p* > 0.05). Pearson correlation test (zero-order correlation) was performed on this study’s continuous data and HRQOL outcomes. The independent samples *t*-test, Mann-Whitney U test, and Kruskal-Wallis H test (one-way ANOVA) were performed on this study’s categorical data to assess the bivariate relationships of HRQOL outcomes with two and three groups of variables. The independent samples *t*-test determined the difference in HRQOL between the correct and incorrect financial literacy groups. A generalised linear model was also employed to assess the relationships of all 10 independent variables with HRQOL (EQ-5D-5L). Dummy variables were formed for categorical variables with more than two groups, which included ethnicity, marital status, education level, and chronic medical condition, along with other ethnicities, separated or divorced, other education levels, and more than one chronic medical condition as the reference categories. All statistical analyses were performed using IBM SPSS (version 23) at 0.05 significance level.

## 3. Results

The self-administered questionnaire survey involved 1186 respondents. The majority of the respondents were male (66.6%). The recorded mean age was 42.10 years (SD of 10.81). Furthermore, most of the respondents were Malay (69.1%) and married (72.7%). About 63.3% of the total respondents reported secondary education as their highest attained education level. The majority of the respondents (73.9%) were employed, and 74.5% of the total respondents reported earning monthly household income of lower than RM 2500. In addition, 74.5% of the total respondents did not have any chronic medical condition. Only 8.0% of the total respondents reported to have more than one chronic medical condition. With 10 as the maximum score, the mean value for financial literacy recorded was 5.95 (1.48). Most of these respondents (67.6%) represented low level of financial literacy. Meanwhile, with 40 as the maximum value, the mean value for financial behaviour recorded was 22.08 (4.79), suggesting respondents’ poor financial behaviour. Additionally, the overall mean value of EQ-5D-5L was 0.96 (0.10).

### 3.1. Association between Independent Variables with EQ-5D-5L Scores

The results of the relationships of all independent variables with EQ-5D-5L are summarised in Table 1. Firstly, the results revealed a negative relationship between age and EQ-5D-5L mean scores. In other words, EQ-5D-5L mean scores decrease as age increase. Secondly, the results demonstrated a significant relationship between marital status and EQ-5D-5L scores. In other words, single individuals in the B40 group had higher EQ-5D-5L scores than married individuals in the B40 group. A similar observation was observed for the relationship between employment status and EQ-5D-5L scores. Meanwhile, the relationship between having chronic medical condition and HRQOL was found to be statistically significant. The results demonstrated that those without any chronic medical condition recorded higher EQ-5D-5L mean scores than those with chronic medical condition. On the contrary, the obtained results demonstrated an insignificant relationship between financial literacy and HRQOL. However, the scores of financial behaviour and EQ-5D-5L were correlated.

### 3.2. Association between Financial Literacy Statements with EQ-5D-5L Scores

Based on the tabulated results in Table 2, most of the respondents in this study provided correct responses to all financial literacy questions, except for the following: (1) “All types of investment in Malaysia are legal” (correct responses: 58.2%); (2) “The Credit Counselling and Debt Management Agency (AKPK) provides loan services” (correct responses: 62.5%). These results revealed significant differences in EQ-5D-5L mean scores in four out of 10 financial literacy questions in favour of those with correct responses, specifically on the use of shopping lists, types of legal investments, wills, and investments.

### 3.3. Multivariate Analysis

Table 3 presents the results of multivariate analysis that involved using a generalised linear model. The results of adjusted model 1 revealed age (β = −0.001, 95% CI = −0.002, −0.001), Malay ethnicity (β = 0.03, 95% CI = 0.003, 0.058), Indian ethnicity (β = 0.041, 95% CI = 0.008, 0.073), and financial behaviour (β = 0.001, 95% CI = 0.0001, 0.003) as significant determinants of HRQOL. The presence of chronic medical condition was then included in the saturated model (adjusted model 2). The results then revealed age (β = −0.001, 95% CI = −0.002, −0.0002) and those without chronic medical condition (β = 0.047, 95% CI = 0.024, 0.07) as significant determinants of HRQOL. In addition, Malay (β = 0.033, 95% CI = 0.006, 0.060) and Indian (β = 0.041, 95% CI = 0.009, 0.073) ethnicities were found to be related to higher HRQOL. After controlling the number of chronic medical condition, the relationship between financial behaviour (β = 0.001, 95% CI = −0.00013, 0.00243) and HRQOL became statistically insignificant. Furthermore, the relationship between financial literacy and HRQOL was found non-existent in this study.

## 4. Discussion

With a sample of 1186 low-income working adults in Malaysia, this study obtained valuable insights on the relationships of financial literacy and financial behaviour with HRQOL during the COVD-19 pandemic. The obtained results demonstrated a significant, negative relationship between age and HRQOL. Both Malay and Indian ethnicities were also revealed to be significant factors that contribute to better HRQOL. Meanwhile, positive financial behaviour was found linked to higher QOL scores in this study. However, with the addition of having chronic medical condition into the model, financial literacy was found unrelated to HRQOL. The results further revealed not being diagnosed with any chronic medical condition as another significant factor that contributes to better QOL. Additionally, the recorded mean score of 0.96 (SD of 0.10) in this study’s EQ-5D-5L, which involved respondents from all regions in Malaysia, was found higher than that of another prior study that similarly involved a population of low socio-economic status in 2016 by Wan Puteh et al. [40].

In particular, the distribution of age in this study was rather even, with the mean age of 42.10 years (SD of 10.81). Ageing has inevitable effects on one’s physical and mental states, which subsequently affect the overall health, well-being, and ultimately, QOL [41,42]. In both models, the negative correlation between age and QOL was found weak, which was found to be in line with the findings of prior studies on a population of low socio-economic status in Malaysia [20]. The findings on the relationship between age and QOL vary according to the tools used to measure QOL—the use of certain tools suggests lower QOL as age increases, with socio-economic factors as the dominant determinants of the variability in QOL [43]. Nonetheless, the weak correlation between age and QOL in this study implied the presence of other more dominant determinants, apart from age, that affect the QOL of low-income working population.

Following that, the results of bivariate analysis revealed a significant relationship between marital status and HRQOL. Widowed and separated/divorced respondents in this study recorded lower HRQOL scores than single and married respondents. Previous studies similarly reported that the QOL of individuals who are either widowed or divorced are affected [44,45]. On the other hand, studies by others reported that single or divorced individuals exhibited a substantially higher likelihood of poor self-rated health [46,47]. Clearly, the influence of marital status on HRQOL should be further explored, especially financially and socially affected single parents during the pandemic [23].

Malaysia is a multi-ethnic and multiracial country. Based on the Population Distribution and Basic Demographic Characteristic Report 2010, the Bumiputera group (73.6%), which includes Malays, indigenous people, and Pribumi of Sabah and Sarawak, represented the majority of the vulnerable B40 population in this country, followed by Chinese (17.5%), Indians (8.5%), and other ethnicities (0.4%) [48]. The results of multivariate analysis in this study revealed both Malay and Indian ethnicities as significant determinants of HRQOL (higher EQ-5D-5L scores). Only a few prior studies similarly focused on the low-income working population in Malaysia, and the findings involving different age groups have remained inconclusive. In a similar study, Sazlina et al. [49] reported that the Indian elderly group recorded lower mental health component of HRQOL. For instance, Loh et al. [50] reported that Malay adolescents recorded lower HRQOL, as compared to adolescents of other ethnicities [34]. In a more recent study, Lai et al. found that the Malay elderly group recorded higher mean control, autonomy, self-realisation, and pleasure (CASP-19) score (than the Chinese and Indian elderly groups), suggesting higher QOL instead [51]. Therefore, it is imperative to further explore these health inequalities across ethnicities in Malaysia for optimal health and well-being. This, in accordance with the United Nation’s SDG, it is imperative to investigate further the differences in HRQOL across different ethnic groups in an effort to reduce inequalities [10].

In addition, several prior studies noted the relationship between employment status and HRQOL (EQ-5D-5L scores) among Korean adults, in which employed adults appeared to have higher QOL than unemployed adults [52,53]. Focusing on the Malaysian context, Boo et al. similarly found that employed adults demonstrated a higher level of life satisfaction [54]. As for the current study, the results of bivariate analysis revealed a significant relationship between employment status and HRQOL, but the results of multivariate analysis revealed otherwise. Wan Puteh et al. similarly found no significant relationship between employment status and QOL (EQ-5D scores) [40].

Similar to other countries, Malaysia experiences productivity loss with the rising cases of chronic medical conditions or also known as non-communicable diseases (NCDs). The health issues related to NCDs encountered by the vulnerable B40 group have substantial impact on their financial and social aspects, as well as HRQOL. The obtained results in the current study demonstrated the impact of the presence of chronic medical condition on QOL. This study’s findings were found consistent with the findings of prior studies on the statistically significant, negative relationship between the presence of chronic medical condition and QOL. Thangiah et al. and Bakar et al. reported similar observations on QOL (in terms of EQ-5D) between those without chronic medical condition and those who were diagnosed with chronic medical condition [20,55].

Notably, the presence of chronic medical condition among the respondents in this study accounted for the relationship between financial behaviour and HRQOL. In other words, poorer financial behaviour resulting from the presence of chronic medical condition contributed to lower QOL among the respondents in this study. However, it should be noted that it was not possible to assign temporarily to this relationship in this cross-sectional study. Nonetheless, it was deemed plausible that those with poorer financial behaviour are more susceptible to NCDs and experience lower QOL. Hence, low-income working individuals with poorer financial behaviour and chronic medical condition represent potentially modifiable risk factors for poorer QOL.

Financial literacy consists of financial, credit, and debt management skills and the knowledge to make financially wise decisions [56]. There have been limited studies on the relationship between financial literacy and HRQOL, which was addressed in the current study. The results of this study revealed low level of financial literacy among most of the respondents. Previous studies that focused on low-income working populations also found similar observations [22,57,58]. Additionally, the results revealed no significant relationship between financial literacy and HRQOL, but individual statement analysis linked knowledge of shopping lists, forms of legal investments, wills, and investments to HRQOL. Given the significance of income as a determinant of QOL, evidence of a low level of financial literacy among low-income populations suggests a cause of concern [59,60,61]. Therefore, it is imperative to explore this particular determinant in order to promote financial literacy and subsequently, increase HRQOL, especially among low-income populations.

Likewise, studies on the relationship between financial behaviour and HRQOL have remained scarce, which was addressed in this study. With the maximum score of 40, the mean score for financial behaviour in this study recorded 22.08 (SD of 4.79), confirming the poor financial behaviour of low-income working population. Financial challenges and financial behaviour are typically linked to poor HRQOL [62,63]. Ahmad et al. found similar results on how low-income earners are linked to low level of financial literacy and poor financial behaviour [57]. On a similar note, Rahman et al. identified financial behaviour as the key predictor of financial well-being, followed by financial stress and financial literacy [23]. Although the Malaysian government has managed to curb the spread of COVID-19, the economic implications of the measures taken by the government, especially the enforcement of MCO, appeared to be more severe than the prior experiences during the Asian financial crisis of 1997–1998 and the global financial crisis of 2008–2009 [64]. The adverse implications of the COVID-19 pandemic on the general population have been rather critical, especially to the B40 group. With HRQOL as a multidimensional concept, the obtained results of this study clearly demonstrated financial behaviour as one of the significant factors that lead to higher QOL. This study successfully proved the gap involving the relationship between financial literacy and financial behaviour. Low levels of both financial factors lead to poorer QOL among the low-income populations.

Considering that individuals with chronic medical condition are more susceptible to experiencing severe symptoms and complications of COVID-19, the Malaysian government introduced more initiatives to reduce the burden of NCDs on the low-income population, including the PEKA B40 National Economic Recovery Plan (PENJANA) that comes with free health screening [65]. The Malaysian government has allocated a substantial number of financial resources for the implementation of these measures and initiatives to mitigate the economic impact of the pandemic, particularly for the vulnerable B40 group [9]. Therefore, it is important to explore the impact of these measures and initiatives on QOL and other related aspects.

Addressing the adverse impact of the pandemic, the Malaysian government released a financial aid package, specifically known as PRIHATIN worth up to RM 250 billion, on 27 March 2020 for all micro, small, and medium-sized businesses to retain their employees. Furthermore, all low-income households with monthly earnings of RM 4000 or less were able to receive incentives of up to RM 1600. The Malaysian government also introduced exemptions on housing and business premise rentals and a moratorium on loan repayment (all loans, except credit card payments) for six months.

### Limitations and Future Studies

This study encountered several limitations. Firstly, this cross-sectional study was not able to obtain adequate evidence to further elucidate the causal relationships of all factors with HRQOL. Furthermore, this study did not consider the influence of the economic changes, such as the financial status during the COVID-19 pandemic, on HRQOL. Therefore, it is recommended for future research to consider conducting a longitudinal study in order to explore this particular scenario closely within a wider timeframe. Secondly, this study’s self-administered questionnaire survey was conducted in selected states of Malaysia due to the large population size in Malaysia. Therefore, it is recommended for future research to obtain a more geographically diverse sample for the purpose of generalisability. It is also suggested to consider an interview-based data collection to address potential reporting bias (through the self-administered questionnaire survey). Lastly, focusing on the low-income working population, this study only examined the influence of age, gender, ethnicity, marital status, education level, employment status, monthly household income, chronic medical condition, financial literacy, and financial behaviour on HRQOL. Therefore, it is suggested for future research to consider other socio-demographic and financial factors in relation to financial well-being.

## 5. Conclusions

The COVID-19 pandemic started in early 2019, and the Malaysian government implemented various measures, including MCO enforcement, to curb the spread of the virus. However, this has negatively affected the vulnerable B40 group. Furthermore, low-income working adults are more likely to take a longer time to recover from their financial losses (due to unemployment or salary cuts). This study presented evidence on how being older and having chronic medical condition can lead to more unfavourable outcomes of HRQOL. Furthermore, the presence of chronic medical condition accounted for the relationship between poorer financial behaviour and lower HRQOL in this study. Therefore, future research should explore how those with chronic medical condition can improve their QOL through interventions that improve financial behaviour.

In addition to the efforts already being made by governments, initiatives such as PeKa B40, mySalam and household living aid (BSH) should be continuously improved and disseminated among the disadvantaged group. By providing opportunities to age actively through financial aid, voluntary activities, community engagement, and improving health and financial literacies, policymakers can mobilise effort to reduce health inequity among all Malaysians. Integrating health promotion and financial literacy in these activities is essential for expanding health and financial security and funnelling funds more efficiently to improve QOL. The policymakers should utilise a comprehensive approach at all levels to improve the lives of many disadvantaged Malaysians particularly among the low-income community.

## Figures and Tables

**Table 1:** Characteristics of productive B40 respondents based on HRQOL (*n* = 1186).

Variables		Mean (SD)	*n* (%)	EQ-5D-5L, Mean (SD)	Pearson’s *r*	*p*-Value
Age (year)	Mean					
	20–30	42.10 (10.81)	226 (19.1)	0.97 (0.09)	−0.131	<0.001 *
	31–40		309 (26.1)	0.97 (0.08)		<0.001 *
	41–50		327 (27.6)	0.95 (0.11)		
	51–60		324 (27.3)	0.93 (0.12)		
Gender	Male		790 (66.6)	0.96 (0.11)		0.198
	Female		396 (33.4)	0.95 (0.09)		
Ethnicity	Malay		820 (69.1)	0.96 (0.10)		0.094
	Chinese		75 (6.3)	0.94 (0.14)		
	Indian		109 (9.2)	0.97 (0.08)		
	Pribumi		115 (9.7)	0.95 (0.10)		
	Others		67 (5.6)	0.93 (0.13)		
Marital Status	Single		166 (14.0)	0.97 (0.07)		0.024 *
	Married		862 (72.7)	0.96 (0.11)		
	Widowed		132 (11.1)	0.93 (0.11)		
	Separated/Divorced		25 (2.1)	0.95 (0.09)		
Education Level	Primary		134 (11.6)	0.94 (0.11)		0.259
	Secondary		731 (63.3)	0.95 (0.11)		
	Pre-University		190 (16.5)	0.97 (0.07)		
	University		90 (7.8)	0.97 (0.08)		
	Others		10 (0.9)	0.98 (0.07)		
Employment Status	Employed		876 (73.9)	0.96 (0.10)		0.010 *
	Unemployed		310 (26.1)	0.94 (0.11)		
Income	Less than RM 2500		883 (74.5)	0.95 (0.11)		0.351
	RM 2500–RM 4850		303 (25.5)	0.96 (0.09)		
Chronic Diseases	None		884 (74.5)	0.97 (0.10)		<0.001 *
	One		207 (17.5)	0.94 (0.10)		
	More than one		95 (8.0)	0.91 (0.12)		
Financial Literacy	Low	5.95 (1.48)	802 (67.6)	0.96 (0.11)	−0.004	0.892
	High		384 (32.4)	0.95 (0.09)		0.716
Financial Behaviour		22.08 (4.79)			0.072	0.013 *
EQ-5D-5L total index				0.96 (0.10)		

* Significant at *p* < 0.05.

**Table 2:** Characteristics of Financial Literacy of productive B40 respondents based on HRQOL (*n* = 1183).

Financial Literacy Statements	*n* (%)	EQ-5D-5L Mean (SD)	*p*-Value
Shopping lists help control spending.			
Wrong	62 (5.2)	0.90 (0.21)	0.042 *
Correct	1121 (94.8)	0.96 (0.09)	
All types of investments in Malaysia are legal.			
Wrong	495 (41.8)	0.95 (0.11)	0.033 *
Correct	688 (58.2)	0.96 (0.10)	
Individuals must save a minimum of 10% of their income.			
Wrong	153 (12.9)	0.95 (0.08)	0.602
Correct	1030 (87.1)	0.96 (0.11)	
All forms of risk are insurable.			
Wrong	416 (35.2)	0.95 (0.09)	0.302
Correct	767 (64.8)	0.96 (0.11)	
A person can distribute all his property through a will.			
Wrong	214 (18.1)	0.94 (0.11)	0.030 *
Correct	969 (81.9)	0.96 (0.10)	
Shariah products are risk-free.			
Wrong	335 (28.3)	0.95 (0.09)	0.241
Correct	848 (71.7)	0.96 (0.11)	
The Credit Counselling and Debt Management Agency (AKPK) provides loan services.			
Wrong	444 (37.5)	0.95 (0.10)	0.442
Correct	739 (62.5)	0.96 (0.11)	
The Central Credit Reference Information System (CCRIS) is a credit bureau that collects, processes, stores, and creates credit information.			
Wrong	252 (21.3)	0.94 (0.11)	0.052
Correct	931 (78.7)	0.96 (0.10)	
Investments with high returns may be high risk.			
Wrong	158 (13.4)	0.94 (0.12)	0.018 *
Correct	1025 (86.6)	0.96 (0.10)	
High inflation means the cost of living is rising rapidly.			
Wrong	150 (12.7)	0.95 (0.10)	0.312
Correct	1033 (87.3)	0.96 (0.11)	

* Significant at *p* < 0.05.

**Table 3:** Multivariate analysis on the determinants of HRQOL.

Factors	Unadjusted			Adjusted Model 1			Adjusted Model 2		
	B	SE	95% CI	B	SE	95% CI	B	SE	95% CI
Age (year)	−0.001 *	0.0003	−0.002, −0.001	−0.001 *	0.0003	−0.002, −0.001	−0.001 *	0.0003	−0.002, −0.0002
Gender			−0.004, 0.021			−0.009, 0.019			−0.110, 0.170
Male	0.008	0.0065		0.005	0.0071		0.003	0.0070	
Female	ref	ref		ref	ref		ref	ref	
Ethnicity									
Malay	0.029 *	0.0132	0.003, 0.055	0.030 *	0.0139	0.003, 0.058	0.033 *	0.014	0.006, 0.060
Chinese	0.010	0.0175	−0.024, 0.044	0.02	0.018	−0.015, 0.056	0.017	0.018	−0.018, 0.052
Indian	0.036 *	0.0162	0.004, 0.067	0.041 *	0.0165	0.008, 0.073	0.041 *	0.016	0.009, 0.073
Pribumi	0.015	0.0167	−0.018, 0.047	0.011	0.0168	−0.022, 0.044	0.015	0.017	−0.018, 0.047
Others	ref	ref		ref	ref		ref	ref	
Marital Status									
Single	0.017	0.0227	−0.028, 0.061	−0.007	0.023	−0.052, 0.039	0.001	0.023	−0.045, 0.046
Married	0.006	0.0215	−0.036, 0.049	−0.004	0.022	−0.047, 0.039	0.001	0.022	−0.042, 0.043
Widowed	−0.019	0.0231	−0.064, 0.027	−0.018	0.023	−0.064, 0.027	−0.013	0.023	−0.058, 0.032
Separated/Divorced	ref	ref		ref	ref		ref	ref	
Employment Status			0.002,0.029			−0.006, 0.023			−0.006,0.022
Employed	0.016 *	0.007		0.008	0.007		0.008	0.007	
Unemployed	ref	ref		ref	ref		ref	ref	
Education Level									
Primary	−0.032	0.0341	−0.099, 0.035	−0.025	0.034	−0.092, 0.042	−0.027	0.0337	−0.093, 0.039
Secondary	−0.023	0.0332	−0.088, 0.042	−0.033	0.033	−0.097, 0.032	−0.03	0.0327	−0.095, 0.034
Pre−University	−0.010	0.0338	−0.076, 0.057	−0.028	0.034	−0.094, 0.038	−0.026	0.0334	−0.091, 0.040
University	−0.011	0.0347	−0.079, 0.057	−0.036	0.035	−0.104, 0.032	−0.036	0.0345	−0.103, 0.032
Others	ref	ref		ref	ref		ref	ref	
Income			−0.021, 0.007						
Less than RM 2500	−0.007	0.007		0.004	0.008	−0.015,0.015	0.003	0.0075	−0.012, 0.018
RM 2500–RM 4850	ref	ref		ref	ref		ref	ref	
Financial Literacy	−0.0001	0.0021	−0.004, 0.004	−0.0005	0.0021	−0.005, 0.004	−0.0005	0.0021	−0.005, 0.004
Financial Behaviour	0.002 *	0.0006	0.001, 0.003	0.001 *	0.0007	0.0001, 0.003	0.001 *	0.0007	−0.00013, 0.00243
Chronic Diseases									
None	0.056 *	0.0113	0.888, 0.931				0.047 *	0.0117	0.024, 0.070
1	0.025 *	0.013	0.034, 0.078				0.021	0.0129	−0.004, 0.047
More than 1	ref	ref					ref	ref	

* Significant at *p* < 0.05.

## Data Availability

All relevant data are within the paper.

## Appendix A. Financial Literacy (Malay Version)/Pengetahuan Kewangan (Versi Melayu)

Pernyataan berikut adalah mengenai literasi kewangan. Tandakan satu kotak yang sesuai sama ada pernyataan berikut adalah SALAH atau BETUL.

*The following statement is about financial literacy. Please tick one box whether the following statement is FALSE or TRUE.*

**No**	**Pernyataan/** ***Statements***	**Salah/** ***Wrong***	**Betul/Correct**
1.	Senarai membeli-belah membantu mengawal perbelanjaan. *Shopping lists help control spending.*	**[····]**	**[····]**
2.	Semua jenis pelaburan di Malaysia adalah sah di sisi undang-undang. *All types of investments in Malaysia are legal.*	**[····]**	**[····]**
3.	Individu perlu menyimpan minimum sebanyak 10% daripada pendapatan. *Individuals must save a minimum of 10% of their income.*	**[····]**	**[····]**
4.	Semua bentuk risiko boleh diinsuranskan. *All forms of risk are insurable.*	**[····]**	**[····]**
5.	Seseorang itu boleh mengagihkan semua hartanya melalui wasiat. *A person can distribute all his property through a will.*	**[····]**	**[····]**
6.	Produk Shariah bebas daripada risiko. *Shariah products are risk-free.*	**[····]**	**[····]**
7.	Agensi Kaunseling dan Pengurusan Kredit (AKPK) memberikan perkhidmatan pinjaman. *The Credit Counselling and Debt Management Agency (AKPK) provides loan services.*	**[····]**	**[····]**
8.	Sistem Maklumat Rujukan Kredit Pusat (CCRIS) merupakan satu biro kredit yang mengumpul, memproses, menyimpan dan mewujudkan maklumat kredit. *The Central Credit Reference Information System (CCRIS) is a credit bureau that collects, processes, stores, and creates credit information.*	**[····]**	**[····]**
9.	Pelaburan dengan pulangan yang tinggi mungkin berisiko tinggi. *Investments with high returns may be high risk.*	**[····]**	**[····]**
10.	Inflasi yang tinggi bermakna kos sara hidup meningkat dengan pesat. *High inflation means the cost of living is rising rapidly.*	**[····]**	**[····]**

## Appendix B. Financial Behaviour (Malay Version)/Tingkah Laku Kewangan (Versi Melayu)

Berasaskan skala di bawah, sila tandakan jawapan yang menerangkan situasi diri anda.

*Based on the scale below, please mark the answer that describes your situation.*

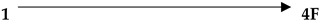

	**Tidak Pernah/*Never* Sangat Kerap/Selalu/*Always***				
**No**	**Pernyataan/Statements**	**Skala/Scale**			
1.	Saya belanja mengikut bajet mingguan atau bulanan.				
	*I spend according to a weekly or monthly budget.*	**1**	**2**	**3**	**4**
2.	Saya mencatat ke mana wang saya dibelanjakan.				
	*I keep track of where my money is spent.*	**1**	**2**	**3**	**4**
3.	Saya mengasingkan wang untuk perbelanjaan kecemasan.				
	*I set aside money for emergency expenses.*	**1**	**2**	**3**	**4**
4.	Saya menyimpan untuk memenuhi matlamat kewangan diri/keluarga.				
	*I save to meet personal/family financial goals.*	**1**	**2**	**3**	**4**
5.	Saya menyimpan resit pembelian.				
	*I keep the purchase receipt.*	**1**	**2**	**3**	**4**
6.	Saya membuat bayaran minimum untuk pinjaman yang dibuat.				
	*I made the minimum payment for the loan made.*	**1**	**2**	**3**	**4**
7.	Saya merekodkan pembayaran ansuran pinjaman.				
	*I recorded the loan instalment payment.*	**1**	**2**	**3**	**4**
8.	Saya lewat membayar ansuran pinjaman.				
	*I’m late paying the loan instalments.*	**1**	**2**	**3**	**4**
9.	Saya meneliti harga barangan dengan berhati-hati sebelum membelinya.				
	*I scrutinize the price of the item before buying it.*	**1**	**2**	**3**	**4**
10.	Saya mempunyai matlamat kewangan jangka masa panjang dan berusaha untuk mencapainya.				
	*I have long-term financial goals and strive to achieve them.*	**1**	**2**	**3**	**4**

## Appendix C. EQ-5D-5L (Malay Version)/EQ-5D-5L (Versi Melayu)

Sila tandakan satu kotak yang menggambarkan keadaan kesihatan anda hari ini dengan paling tepat.

*Under each heading, please tick one box that best describes your health today.*

**PERGERAKAN/MOBILITY**	
Saya tidak menghadapi masalah untuk berjalan	**[···]**
*I have no problems in walking about*	**[···]**
Saya menghadapi sedikit masalah untuk berjalan	**[···]**
*I have slight problems in walking about*	**[···]**
Saya menghadapi masalah yang sederhana untuk berjalan	**[···]**
*I have moderate problems in walking about*	**[···]**
Saya menghadapi masalah yang teruk untuk berjalan	**[···]**
*I have severe problems in walking about*	**[···]**
Saya tidak berupaya untuk berjalan	**[···]**
*I am unable to walk about*	**[···]**
**PENJAGAAN DIRI/SELF-CARE**	
Saya tidak menghadapi masalah untuk membersihkan diri atau memakai	**[···]**
sendiri pakaian saya	**[···]**
*I have no problems washing or dressing myself*	
Saya menghadapi sedikit masalah untuk membersihkan diri atau memakai	**[···]**
sendiri pakaian saya	**[···]**
*I have slight problems washing or dressing myself*	
Saya menghadapi masalah yang sederhana untuk membersihkan diri atau	**[···]**
memakai sendiri pakaian saya	**[···]**
*I have some problems washing or dressing myself*	
Saya menghadapi masalah yang teruk untuk membersihkan diri atau memakai	**[···]**
sendiri pakaian saya	**[···]**
*I have severe problems washing or dressing myself*	
Saya tidak berupaya untuk membersihkan diri atau memakai sendiri pakaian saya	**[···]**
*I am unable to wash or dress myself*	**[···]**
**AKTIVITI-AKTIVITI BIASA (misalnya bekerja, belajar, membuat kerja rumah, aktiviti-aktiviti keluarga atau masa lapang)/** **USUAL ACTIVITIES (e.g., work, study, housework, family or leisure activities)**	
Saya tidak menghadapi masalah untuk melakukan aktiviti-aktiviti biasa saya	**[···]**
*I have no problems doing my usual activities*	**[···]**
Saya menghadap sedikit masalah untuk melakukan aktiviti-aktiviti biasa saya	**[···]**
*I have slight problems doing my usual activities*	**[···]**
Saya menghadapi masalah yang sederhana untuk melakukan aktiviti-aktiviti biasa saya	**[···]**
*I have some problems doing my usual activities*	**[···]**
Saya menghadapi masalah yang teruk untuk melakukan aktiviti-aktiviti biasa saya	**[···]**
*I have severe problems doing my usual activities*	**[···]**
Saya tidak berupaya untuk melakukan aktiviti-aktiviti biasa saya	**[···]**
*I am unable to do my usual activities*	**[···]**
**KESAKITAN/KETIDAKSELESAAN/PAIN/DISCOMFORT**	
Saya tidak berasa sakit atau tidak selesa	**[···]**
*I have no pain or discomfort*	**[···]**
Saya berasa sakit atau tidak selesa sedikit	**[···]**
*I have slight pain or discomfort*	**[···]**
Saya berasa sakit atau tidak selesa yang sederhana	**[···]**
*I have moderate pain or discomfort*	**[···]**
Saya berasa sakit atau tidak selesa yang teruk	**[···]**
*I have severe pain or discomfort*	**[···]**
Saya berasa sakit atau tidak selesa yang teramat sangat	**[···]**
*I have extreme pain or discomfort*	**[···]**
**ANXIETY/DEPRESSION**	
Saya tidak berasa risau atau murung	**[···]**
*I am not anxious or depressed*	**[···]**
Saya berasa risau atau murung sedikit	**[···]**
*I am slightly anxious or depressed*	**[···]**
Saya berasa risau atau murung yang sederhana	**[···]**
*I am moderately anxious or depressed*	**[···]**
Saya berasa risau atau murung yang teruk	**[···]**
*I am severely anxious or depressed*	**[···]**
Saya berasa risau atau murung yang teramat sangat	**[···]**
*I am extremely anxious or depressed*	**[···]**
